# Improvements in Polio Vaccination Status and Knowledge about Polio Vaccination in the CORE Group Polio Project Implementation Areas in Pastoralist and Semi-Pastoralist Regions in Ethiopia

**DOI:** 10.4269/ajtmh.19-0022

**Published:** 2019-10

**Authors:** Fasil Tessema, Filimona Bisrat, Legesse Kidane, Muluken Assres, Tenager Tadesse, Bethelehem Asegedew

**Affiliations:** 1 Department of Epidemiology, Faculty of Public Health, Jimma University, Jimma, Ethiopia;; 2 CORE Group Polio Project/Ethiopia, Addis Ababa, Ethiopia

## Abstract

Strengthening routine immunization is one of the four prongs of the Global Polio Eradication Initiative. Achieving this requires improving immunization coverage in hard-to-reach areas. The objectives of this analysis were to assess levels of oral polio vaccination coverage and challenges in pastoral and semi-pastoral regions in Ethiopia. The analysis included vaccination-related data for children aged 12–23 months from the 2011 Ethiopian Demographic and Health Survey (EDHS) and from surveys carried out by the CORE Group Polio Project (CGPP) in 2013, 2015, and 2017. The EDHS data were from the entire regions (states) of Somali; Oromia; Southern Nations, Nationalities, and Peoples; Benshangul-Gumuz; and Gambella, whereas the CGPP data were for portions of these states where the CGPP was working and consisted entirely of pastoralist or semi-pastoralist populations. The overall polio immunization coverage rate showed upward trend from 39.6% in the 2011 EDHS to 72.6% for 2017 survey of children in the CGPP intervention areas. The evidence suggests that the CGPP was able to achieve increasing levels of coverage in the hardest-to-reach areas of these states and that the levels were higher than those achieved in the states as a whole. The strategies used by the CGPP/Ethiopia to increase coverage appear to have been effective. Other characteristics associated with full polio immunization included mother’s religion and education, whether the mother had heard about polio, knowledge on the effect of many polio vaccine doses, and age at first polio immunization.

## INTRODUCTION

Ethiopia’s last indigenous case of wild poliovirus occurred in 2001, and its last case of imported wild poliovirus was reported in 2014.^1^ Ethiopia remains a high-priority country in the Global Polio Eradication Initiative (GPEI) because of the high risk of importation of wild poliovirus from the neighboring countries of Kenya, Djibouti, and especially from the conflict-affected countries of Somalia and South Sudan.^2^ In addition, Ethiopia is also a high-priority country for the GPEI because of its large population of highly dispersed and often mobile people living in areas of low population density, making high levels of immunization coverage a challenge. In the most recent Ethiopian Demographic and Health Survey (EDHS) published in 2016, only 30% of children living in rural areas in Ethiopia were fully vaccinated at the appropriate ages. Furthermore, only 53% of children in rural areas had received the full recommended three doses of oral polio vaccine (OPV 3).^3^

**Figure 1.**
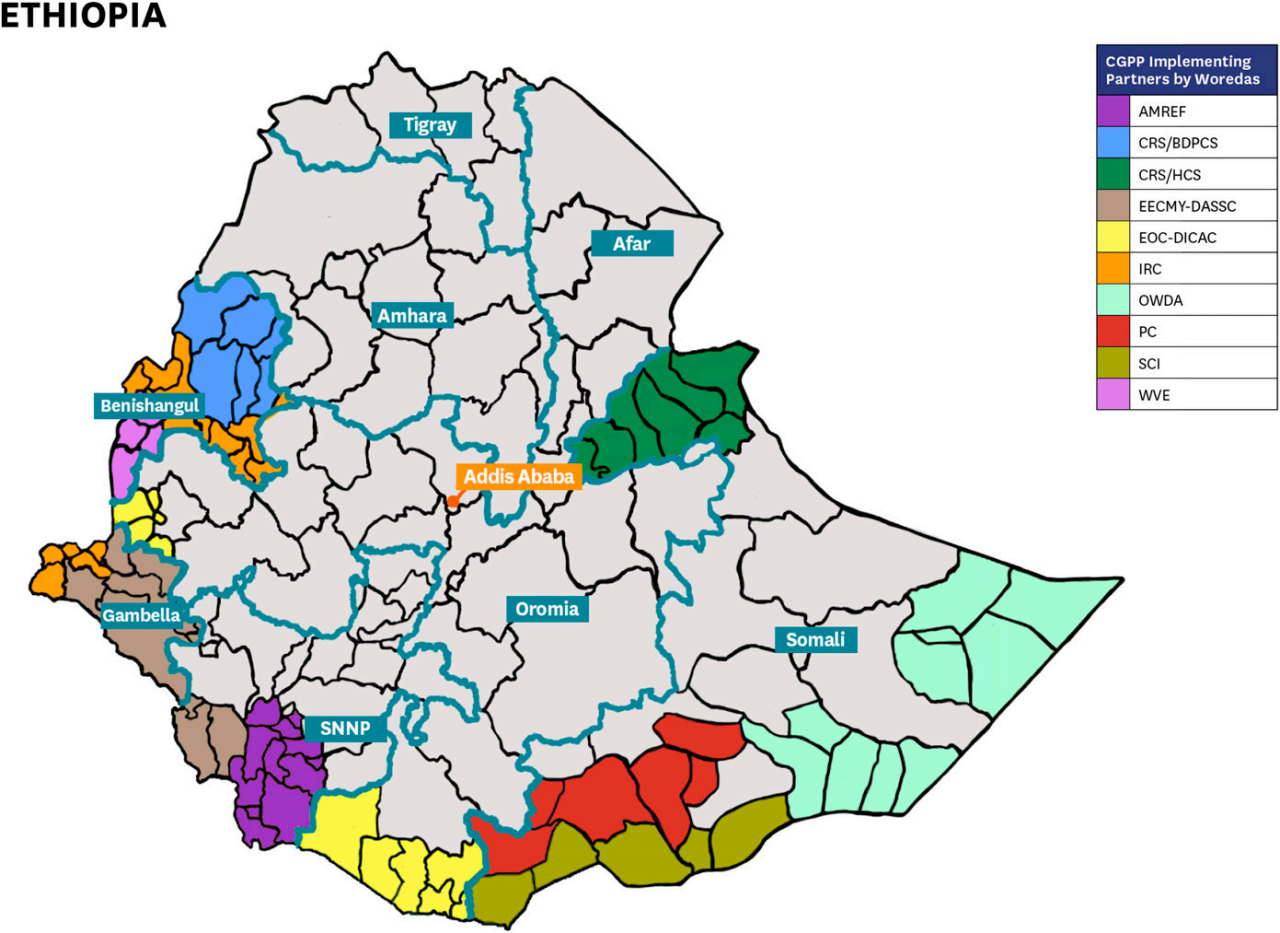
CORE Group Polio Project/Ethiopia implementation districts and non-governmental organization partners in Ethiopia.^4^

The CORE Group Polio Project (CGPP) has been active in Ethiopia since 2001, focusing on promotion of routine immunization services, polio immunization in particular, and surveillance for vaccine-preventable diseases in hard-to-reach pastoralist and semi-pastoralist populations and in high-risk border areas of Ethiopia in the following regional states: Somali; Oromia; Southern Nations, Nationalities, and Peoples (SNNP); Benshangul-Gumuz; and Gambella Region (Figure 1). A history of the CGPP and its work in Ethiopia are described elsewhere in this series.^2^ The CGPP implements its programs through a network of international and national non-governmental organizations coordinated by a national secretariat.

This article examines the population coverage of OPV in the CGPP program areas and the influences on coverage, using survey data collected between 2011 and 2017. The CGPP program areas contain 13 million children younger than 15 years, and the CGPP has trained more than 12,000 community volunteers (CVs) to promote immunizations and conduct surveillance in the CGPP program areas (described elsewhere in this series^5^).

## METHODS

### Data sources, sampling methods, and data collection procedures.

Data for this analysis were extracted from five household surveys that were conducted in the CGPP implementation area: three CGPP surveys that were conducted in 2013, 2015, and 2017, and the 2011 and 2016 EDHS region-wide data for those regions in which the CGPP is operating. The CGPP contracted with an independent consultant from Addis Ababa for the mid-term (2015) survey and a consultant from the Faculty of Public Health at Jimma University for the baseline (2013) and final evaluation (2017) surveys. The data from the 2011 to 2016 EDHS survey used for the analysis were obtained from Measure DHS. The sampling procedure for the 2011 and 2016 EDHS survey is described elsewhere.^3,6^ The EDHS data used for this analysis are representative of the entire regions where the CGPP operates, whereas the CGPP survey data are representative only of those portions of the regions where CGPP operates.

The sampling procedure for the three CGPP surveys was identical: the standard WHO 30-cluster sampling methodology, but the sample size was expanded to compare differences between geographic areas within the CGPP program area. The sample size for specific zones within each region where the CGPP was working was determined by a probability-proportional-to-size (PPS) method. The sample sizes were as follows: 607 for the 2013 survey, 585 for the 2015 survey, and 672 for the 2017 survey.

### Data analysis.

Only a subset of the 2011 and 2016 EDHS survey data was used for this study, namely, the data obtained from households in the regional states where the CGPP is operating: Somali, Oromia, SNNP, Benshangul-Gumuz, and Gambella and children aged 12–23 months. The CGPP implementation areas include five of nine zones of Somali, all three zones in Benshangul-Gumuz, all three zones in Gambella, two of 20 zones in Oromia, and two of 21 in SNNPR. All of these zones are located in pastoral and semi-pastoral regions that are considered hard-to-reach areas or in high-risk border areas (Figure 1).

In all five surveys, data were collected from mothers during face-to-face household interviews. The respondents for the three CGPP surveys were the mothers or caregivers (hereafter referred to as mothers) of children 12–23 aged months, whereas the 2011 and 2016 EDHS survey collected childhood immunization data from mothers of children younger than 5 years. For our analysis, we extracted data of children aged 12–23 months from the EDHS surveys to match the analysis of CGPP survey category. Sociodemographic variables related to mothers and children were coded similarly across surveys before analysis. During the 2013 CGPP baseline survey, mother’s age, gender of child, birth date, place of birth, and OPV 2 status were not collected. Place of birth was also not collected in the 2015 survey. Thus, these data are missing from the tables in the results section.

Oral polio vaccination coverage was calculated based on information written on the child’s vaccination card if present or, if not, on a report from the mother. For the calculation of OPV coverage, weights were used for the two EDHS surveys, whereas for CGPP surveys, no weight was used as the sample was carried out using PPS, but the standard errors for the cluster design were adjusted using the “svy” command in STATA. A child was considered fully vaccinated for polio if all three doses of OPV (OPV 1, OPV 2, and OPV 3) were documented to have been received irrespective of whether the birth dose (OPV 0) had been received. Age at vaccination was determined for polio birth doses and for the first dose (OPV 1) provided that the date of birth and the date of vaccination were available. Those children who received OPV 0 within the first 2 weeks of birth and those who received OPV 1 at 6 weeks of age or later were considered timely vaccinated. In addition, the time intervals between OPV 1, OPV 2, and OPV 3 doses were calculated for those children whose dates of vaccination were recorded on their vaccination card. An interval of fewer than 28 days was considered as early vaccinated.

We used STATA version 14.0 (Stata Corp., College Station, TX) for analysis of the data and the chi-squared test of association between individual background characteristics and the five separate surveys. To assess the independent influence of specific background factors on full polio immunization, we used multivariable binary logistic regression and calculated adjusted odds ratios (ORs) and their associated 95% confidence intervals using only the data from the three CGPP surveys.

### Ethical considerations.

Measure DHS gave permission for analysis of the 2011 and 2016 EDHS data. As the CGPP survey data were collected for monitoring and evaluation purposes rather than for research purposes, we did not request formal approval from an institutional review board to carry out the surveys in 2013, 2015, and 2017. Nonetheless, before the implementation of the surveys, the CGPP secretariat in Addis Ababa provided each of the zonal and regional health offices with an official letter stating the purpose of the surveys. We obtained verbal approval from all respondents after informing them that their participation was voluntary and that their responses would be kept strictly confidential.

## RESULTS

### Comparison of background characteristics for respondents in the CGPP implementation area with those in the regional states as a whole.

Table 1 shows the background characteristics of sampled household respondents and children included in different surveys. Distribution of the sampled population by region of residence, urban versus rural location of residence, age, education and religion of mother, and gender of child are all similar in the three CGPP surveys. Somali households comprise 38–40% of the respondents in the CGPP surveys, whereas they comprise only 15% of the respondents in the subset of 2011 EDHS data used for our analysis. The characteristics of the respondents in the CGPP surveys were otherwise broadly similar to those in the 2011 EDHS data included in our analysis except that the CGPP surveys contained a slightly higher percentage of Muslims (48–52% compared with 43% in the DHS data). Of note, however, is that the 2011 EDHS report indicates that 90% of respondents delivered their baby at home, and the percentage of home deliveries declined to 73% in 2016; the 2017 CGPP survey data indicated a much smaller percentage of home deliveries—47%. The lower percentage of home deliveries in the CGPP areas may reflect the fact that one of the core activities of CVs is the promotion of facility deliveries among pregnant mothers. Across all the five surveys, respondents were overwhelmingly rural (87–95%) and had limited education (64–67% had no education).

**Table 1:** Distribution of survey respondents by region, urban/rural location, respondent age and educational status, gender of child, and place of child’s birth, by survey

Characteristics	Survey										Chi-squared *P*-value
	EDHS 2011		EDHS 2016		CORE Group Polio Project Survey						
					2013		2015		2017		
	*n*	%	*n*	%	*n*	%	*n*	%	*n*	%	
Region											< 0.001
Oromia	284	28.6	285	27.9	61	10.0	105	17.9	111	16.5	
Somali	145	14.6	217	21.3	232	38.2	233	39.8	271	40.3	
Benshangul-Gumuz	168	16.9	156	15.3	100	16.5	98	16.8	100	14.9	
Southern Nations, Nationalities, and Peoples’ Region	250	25.2	228	22.4	154	25.4	109	18.6	110	16.4	
Gambella	147	14.8	134	13.1	60	9.9	40	6.8	80	11.9	
Residence											< 0.001
Rural	876	88.1	128	20.8	570	93.9	552	94.7	582	86.6	
Urban	118	11.9	892	79.2	37	6.1	31	5.3	90	13.4	
Age of mother											< 0.001
< 20	56	5.6	52	5.1	–	–	28	4.8	51	7.6	
20–29	542	54.5	533	52.4	–	–	362	62.1	391	58.2	
30–39	328	33.0	381	37.4	–	–	179	30.7	205	30.5	
40+	68	6.8	54	5.3	–	–	14	2.4	25	3.7	
Mother’s education											< 0.001
No education	663	66.7	656	64.3	395	65.1	398	68.3	433	64.4	
1–4 grade	184	18.5	146	14.3	78	12.9	22	3.8	56	8.3	
5–8 grade	114	11.5	136	13.3	92	15.2	53	9.1	107	15.9	
9 or above	33	3.3	82	8.0	42	6.9	110	18.9	76	11.3	
Mother’s religion											< 0.001
Muslim	430	43.3	508	49.8	293	48.3	299	51.1	347	51.6	
Christian*	518	52.1	472	46.3	221	36.4	245	41.9	286	42.6	
Other†	46	4.6	40	3.9	93	15.3	41	7.0	39	5.8	
Total	994	100.0	1,020	100.0	607	100.0	585	100.0	672	100.0	
Gender of the child											0.235
Male	513	51.6	506	49.6	–	–	286	49.1	362	53.9	
Female	481	48.4	514	50.4	–	–	297	50.9	310	46.1	
Place of birth of child											< 0.001
Home	898	90.3	690	67.6	–	–	–	–	315	47.4	
Health facility	96	9.7	330	32.4	–	–	–	–	350	52.6	
Total	994	100.0	1,020	100.0	–	–	–	–	665	100.0	–

* Christian includes Orthodox Christians, Protestants, and Catholics.

† “Other” includes those with a traditional religion or no religion. EDHS = 2011 Ethiopian Demographic and Health survey. “–” indicates that no data were collected.

### Comparison of oral poliovirus immunization coverage levels in the CGPP implementation area with those in the regional states as a whole based on information from card and mothers’ report.

**Figure 2.**
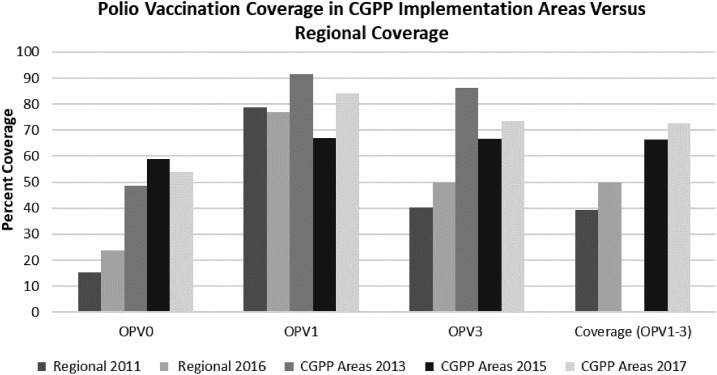
Dose-specific and complete oral polio vaccine (OPV) coverage levels in regional states in 2011 and 2016 and in the CGPP implementation areas in 2013, 2015, and 2017. Data for OPV 2 were not collected in 2013.

Figure 2 compares the levels of OPV immunization coverage for the three CGPP surveys in the high-risk and hard-to-reach CGPP implementation areas with those for the entire populations of the five regional states where the CGPP implementation areas are located. Oral polio vaccine 0 coverage is two to three times higher in the CGPP area compared with regional values (49–59% versus 15–27%). OPV1 and OPV2 immunization coverage levels are comparable (data for OPV 2 were not available for 2013 baseline survey), and OPV 3 levels are approximately two times higher (67–86% versus 41%) in the CGPP areas. Full OPV coverage, based on appropriate dosing intervals, is substantially higher in the CGPP intervention areas than in the regional states as a whole (66–73% versus 40%). Although the OPV coverage levels in the CGPP implementation area did not show any consistent improvements between 2013 and 2017, levels remained steady and were consistently much higher than for the regional states as a whole. The data used to calculate coverage are shown in Supplemental Appendix Table 1.

The other indicators were age at OPV 0 and OPV 1, and the interval between successive doses of OPV (i.e., the interval between OPV 1 and 2, and between OPV 2 and 3). Although OPV 0 is supposed to be provided within the first 15 days of life, 14.6% of children were given OPV 0 after 15 days of birth. There was improvement in this indicator over time, with a declining trend from 2011 (58.7%) to 2017 (9.5%) in the percentage of children in the CGPP program areas receiving their OPV 0 vaccination after 15 days of life. Similarly, 23.6% of children took OPV 1 before the minimum starting age of 6 weeks of age, with the highest percentage (28.2%) observed in 2015. In addition, the minimum interval between successive doses of OPV of at least 28 days was not respected in a substantial percentage of cases: the percentage of children who were vaccinated with OPV 2 less than 4 weeks after their vaccination with OPV 1 was 9.8% according to the dates shown in the vaccination cards in the two CGPP surveys (2015 and 2017), and 7.5% received OPV 3 before a sufficient time interval had elapsed after OPV 2 (see Supplemental Appendix Table 1).

### Influences on OPV coverage.

An analysis of the influences on OPV coverage indicate that coverage levels are slightly higher among mothers aged 20–29 years compared with younger and older mothers for the CGPP implementation areas (see Supplemental Appendix Figure 1). Oral polio vaccine coverage levels are lower among mothers with no education compared with those with formal education, but coverage does not increase among those with at least some education as the educational level increases (see Supplemental Appendix Figure 2). Religion had a modest influence on OPV coverage. Mothers who are Christian have children with slightly higher coverage levels than do those who are Muslim or who are in the “Other” religious category (see Supplemental Appendix Table 2).

**Figure 3.**
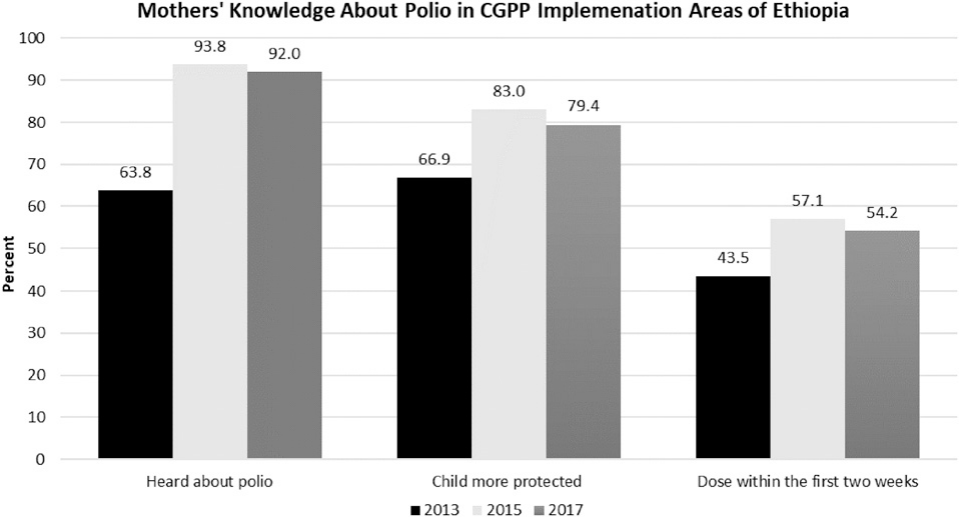
Mothers’ knowledge about polio, the effect of multiple polio vaccine doses on children, and age at first polio vaccination in pastoral and semi-pastoral hard-to-reach areas in Ethiopia, CGPP 2013–2017.

The influence of background characteristics on the knowledge of mothers about polio, on their opinion of the effect of many OPV doses on children, and on the age at first dose of OPV were assessed for the respondents in each of the three CORE Group surveys. As shown in Figure 3, there is a notable and sustained increase between 2013 and 2017 in the percentage of mothers who had heard about polio, who believed that their child would be more protected with multiple doses of polio vaccine, and who knew that children should receive their first polio vaccination during the first 2 weeks of life. Knowledge was consistently better for mothers in urban compared with rural areas, for those with more education, for Muslims, and for those whose child had been born in a health facility (see Supplemental Appendix Table 3).

About 92% of the respondents in the CGPP implementation areas had heard about polio and acute flaccid paralysis (AFP) in 2017 compared with 64% in 2013. Similar marked improvements in knowledge were noted between 2013 and 2015 and sustained in 2017 for awareness about the benefits of multiple OPV doses (i.e., it provides more protection) and knowledge about when her child should receive its first polio vaccination (during the first 2 weeks of life) (Figure 3). During the 2017 survey, source of information on polio was inquired for those respondents who reported that they had heard about polio. Accordingly, among those who had heard about polio or AFP (617), health workers (462 [74.9%]), CVs (362 [58.7%]), community leaders (143 [23.2%]), and friends and neighbors (143 [23.2%]) were the most reported sources of information.

### Multivariate analysis.

We fitted a multivariable binary logistic regression model for children who received the third polio vaccine dose (OPV 3), verified from card, to identify independent factors that influenced the uptake of OPV coverage, combining data from the three CGPP surveys (2013, 2015, and 2017) after considering the design effect of the cluster sampling. The important statistically significant independent influences on OPV 3 coverage, as shown in Table 2, were survey year (time), mothers’ religion and educational status, and mothers’ belief on the effect of repeated polio vaccination. There was an improvement in coverage over time in 2015 compared with the baseline in 2013 after controlling for other independent variables. Christian mothers had children with a higher rate of OPV 3 vaccination (OR = 2.67) compared with Muslim mothers. Mothers with 5–8 and 9+ years of education had higher levels of OPV 3 immunization (OR = 2.02 and OR = 3.90, respectively) compared with mothers with no education. Those who knew that many doses of OPV would provide additional protection to the child and do not know the effect had a higher rate of OPV 3 coverage compared with those who said it would harm the child (OR = 3.57 and OR = 2.66, respectively).

**Table 2:** Independent factors associated with the third dose of oral polio vaccination coverage* in CORE Group Polio Project implementation areas in pastoral and semi-pastoral regions in Ethiopia, 2013–2017

Factors	OR	*P*-value	95% CI for OR	
Survey (CORE 2013)				
CORE 2015	2.78	0.006	1.35	5.73
CORE 2017	1.70	0.120	0.87	3.35
Mother’s religion (Muslim)				
All Christians	2.67	0.001	1.48	4.82
Others†	1.06	0.848	0.60	1.86
Mother’s education (No education)				
1–4 grade	1.01	0.980	0.51	1.98
5–8 grade	2.02	0.002	1.31	3.13
9 or above	3.90	0.000	2.15	7.07
Effects of repeated oral polio vaccine (harmed the child)				
Child more protected	3.57	0.001	1.76	7.25
Child not helped or harmed	0.68	0.605	0.15	3.00
Don’t know/not sure	2.66	0.042	1.04	6.84
Constant	−5.15	0.000	0.04	0.24

OR = odds ratio.

* Oral polio vaccine 3 coverage was calculated based on information only from child vaccination card.

† Others include those with traditional beliefs and those who reported that they have no religion.

## DISCUSSION

Our analysis included five data sets: the 2011 and 2016 EDHS survey (which included data only from the five regions, which contained the CGPP implementation regions), and subsequent surveys conducted by CORE Group in 2013, 2015, and 2017 in the pastoral and semi-pastoral hard-to-reach areas in Somali, Gambella, Benshangul-Gumuz, Oromia, and SNNPR where the CGPP was implementing its activities. The population coverage of polio vaccination was substantially higher in the CGPP implementation areas compared with the regions as a whole. The birth polio vaccine (OPV 0) coverage increased 3-fold and the coverage of the third polio vaccine dose (OPV 3) almost doubled from 2011 to 2017. Based on information from vaccination cards in the CGPP areas, the multivariate analysis demonstrated that OPV 3 coverage had a statistically significant associations with the date of the survey (coverage increased over time), mothers’ religion (higher coverage among children whose mothers were Christian), and education (higher coverage among children whose mothers had more education) as well as mothers’ who believe that multiple doses are beneficial and provide more protection.

### Oral polio vaccination coverage and factors.

Strengthening routine immunization is one of the four prongs of the GPEI. The remaining road toward eradication will require improved access to populations in hard-to-reach areas.^7^ The objectives of this analysis were to assess levels of oral polio vaccination coverage and challenges in pastoral and semi-pastoral regions in Ethiopia.

Survey data from 2015 to 2017 (based on card plus maternal report) showed oral polio vaccination coverage for the three doses (OPV 1, OPV 2, and OPV 3) in pastoral and semi-pastoral region of Ethiopia was 74.2%; 17.0% had never received any OPV. Birth dose OPV (OPV 0) coverage for these areas estimated from the 2013, 2015, and 2017 surveys was 54.1%. The 2016 EDHS report indicated that for the national as a whole, 16% of children aged 12–23 months had not received any polio vaccinations, 81% had received their first dose of polio vaccine, and 56% received three doses of polio vaccine.^3^ A study conducted in Jigjiga woreda, Somali Region, reported OPV 0 and OPV 3 coverage, based on card and history, of 14.6% and 44.1%,^8^ and the 2011 EDHS reported an OPV 3 coverage of 44.3%,^6^ lower than our findings for the CGPP implementation area. Arba Minch, in southern Ethiopia, reported a very high OPV 3 coverage of 86.3%.^9^

Full OPV coverage was significantly and independently associated with survey year, maternal education and religion, whether mothers had heard about polio, their knowledge about the effect of many OPV doses, and the starting age of first dose of polio vaccine. These results are consistent with other studies. The 2016 EDHS reported that vaccination coverage increases with mother’s education,^3^ whereas Kiptoo et al.^10^ found that lack of knowledge on immunization schedule, nomadic life style, low education level, and home delivery were significant factors associated with low immunization coverage in Kenya. Similarly, analysis of the Pakistan DHS 2012–2013 data identified maternal education as one of the factors significantly associated with complete OPV vaccination.^11^ A similar independent association between religion and vaccination status was reported in a study identifying factors associated with full immunization in Techiman Municipality, Ghana.^12^

Although the high prevalence of home deliveries contributes to low OPV 0 coverage,^13^ another reason for low OPV 3 coverage might be the high dropout rate between OPV 1 and OPV 3. Although the national OPV dropout rate was substantial according to the 2011 EDHS (48.3%),^6^ in the CGPP implementation areas it was 13.7% or less in the 2013, 2015, and 2017 CGPP surveys. Notably, the average dropout rate over the period from 2013 to 2017 in the CGPP implementation areas was 7.3%, which is far lower than in the national 2011 and 2016 EDHS surveys.

Other studies have reported varying dropout rates. In Kenya^10^ and Angola,^13^ relatively higher dropout rates have been reported (19.1% and 33.0%, whereas a study conducted in the urban slums of Jagdalpur city, India, reported a dropout rate of only 8.9%,^14^ similar to our findings. The CGPP programming has focused on ensuring that children in its program implementation areas receive all doses of OPV. The low dropout rate reported for the CGPP implementation area suggests that these efforts have made a difference and should continue.

### Vaccination timing and interval.

The vaccination series for OPV should be completed as early in life as possible as the age at highest risk of wild poliovirus infection in most developing countries occurs between 6 and 24 months of age. Currently, the recommended schedule for OPV is at birth, 6, 10, and 14 weeks of age. OPV administration at the recommended ages and in accordance with the recommended intervals between doses provides optimal protection.^15^ The results from this study indicate that a substantial percentage of children are not receiving OPV doses with the prescribed spacing or at the prescribed age. In this regard, our analysis of the CGPP 2015 survey showed that 19.8% of children received OPV 0 after 15 days of age and 25.4% received OPV 1 before 6 weeks of age. The 2017 CGPP survey data showed that 9.1% received OPV 0 after 15 days of age and 17.3% received OPV 1 before 6 weeks of age. In addition, an analysis of the time interval between successive doses of OPV indicated that 11.9% and 7.0% of children received OPV 2 less than 4 weeks after receiving OPV 1 in 2015 and 2017, respectively. Similarly, 5.8% and 8.1% of children received OPV 3 less than 4 weeks after receiving their dose of OPV 2 in 2015 and 2017, respectively. However, these rates of inappropriate timing of doses are significantly lower than those reported in the 2011 EDHS. Although the issues of timeliness and spacing are not the ones that plague Ethiopia alone, as studies in Uganda and Nigeria demonstrate,^16,17^ they are nonetheless notable. Doses administered too close together or at a too young age (after the birth dose) can lead to a suboptimal immune response.^15^ The results of our study suggest that providers need to take more care in ensuring that polio immunizations are given at appropriate times.

## CONCLUSION

Although significant gains have been made in polio-related vaccination coverage in pastoral and semi-pastoral regions of Ethiopia, rates remained below the desirable coverage of 80–90%. Mothers’ knowledge about polio immunization, their religion, and educational level are important predictors of full polio vaccination status in their children. These findings point to the importance of educating young girls and women and ensuring access to primary and secondary schooling. Continuous polio awareness creation activities need to consider the local context and the involvement of local stakeholders, including community leaders and volunteers, and providing special training to CVs to provide birth dose OPV for home deliveries may be an area that might be put in place in hard-to-reach area in collaboration with community health workers. Polio-related education must include mothers with low or no education, as their children are at risk of incomplete vaccinations. Overall, almost all (85%) mothers in the CGPP surveys had heard of polio. However, more than a quarter had misconceptions about the benefits of more polio doses, and 48% wrongly stated the starting age for polio vaccine. Education should focus on increasing knowledge of vaccine benefits and the importance of timeliness in dosing.

In addition, this study found that children of Muslim mothers were less likely to be vaccinated against polio. It is important to understand the factors that underlie this finding. The CGPP should continue to work with religious leaders, both Muslim and Christian, to dispel any misconceptions linked to religious teachings and strengthen accurate knowledge among Muslim mothers.

Vaccination cards hold critical information about vaccination types, dates, and spacing. Without them, it is difficult to get accurate information. Survey data indicated that many caregivers did not have vaccination cards accessible or that they were possibly inaccurately filled out. The CGPP should continue to support parents in obtaining and retaining vaccination cards. Training and refresher training for health workers and immunization providers is necessary to improve quality and accuracy of recording. This is necessary to ensure that children are given needed vaccines and that they are given in a timely manner.

Ethiopia has made great, notable strides in its efforts to increase polio vaccination coverage over the last 10 years. The CGPP has leveraged community networks and partnerships in the most hard-to-reach, highest risk areas of the country. These efforts have made it possible to increase polio vaccination coverage rates and to decrease dropout rates. Improving the timeliness of polio immunization administration requires continued emphasis.

## Supplemental materials, tables, and figures

Supplemental materials

## References

[1] GPEI, 2015 Ethiopia Successfully Interrupts Wild Polio Virus Transmission. Available at: https://afro.who.int/news/ethiopia-successfully-interrupts-wild-polio-virus-transmission. Accessed January 1, 2019.

[2] Losey L 2019 The CORE Group Polio Project: an overview of its history and its contributions to the global polio eradication initiative. Am J Trop Med Hyg 101 (Suppl 4): 4–14. 10.4269/ajtmh.18-0916 PMC6776098 31760971

[3] Ethiopia Central Statistical Agency, ICF International, 2016 Ethiopia Demographic and Health Survey 2016. Available at: https://www.dhsprogram.com/pubs/pdf/FR328/hFR328.pdf. Accessed December 26, 2018.

[4] Stamidis K Bologna L Losey L, 2018 CORE Group Polio Project (CGPP) Final Evaluation Report 2017. Available at: https://coregroup.org/wp-content/uploads/2018/06/CGPP-Evaluation-Report-FINAL-5-10-2018.pdf. Accessed January 1, 2019.

[5] Asegedew B Tessema F Perry H Bisrat F, 2019 The CORE Group Polio Project's community volunteers and polio eradication in Ethiopia: self-reports of their activities, knowledge, and contributions. Am J Trop Med Hyg 101 (Suppl 4): 45–51. 31760977 10.4269/ajtmh.18-1000 PMC6776091

[6] Ethiopia Central Statistical Agency, ICF International, 2012 Ethiopia Demographic and Health Survey 2011 Available at: https://www.usaid.gov/sites/default/files/documents/1860/Demographic%20Health%20Survey%202011%20Ethiopia%20Final%20Report.pdf. Accessed January 1, 2019.

[7] Parry NM, 2017 Despite Progress Towards Global Eradication of Polio, Measles, & Rubella, Challenges Remain. Available at: http://www.contagionlive.com/news/despite-progress-towards-global-eradication-of-polio-measles-and-rubella-challenges-remain. Accessed January 1, 2019.

[8] Mohamud AN Feleke A Worku W Kifle M Sharma HR, 2014 Immunization coverage of 12–23 months old children and associated factors in Jigjiga district, Somali National Regional State, Ethiopia. BMC Public Health 14: 865. 25146502 10.1186/1471-2458-14-865 PMC4158082

[9] Animaw W Taye W Merdekios B Tilahun M Ayele G, 2014 Expanded program of immunization coverage and associated factors among children age 12–23 months in Arba Minch town and Zuria District, southern Ethiopia, 2013. BMC Public Health 14: 464. 24884641 10.1186/1471-2458-14-464 PMC4032449

[10] Kiptoo E Esilaba M Kobia G Ngure R, 2015 Factors influencing low immunization coverage among children between 12–23 months in East Pokot, Baringo country, Kenya. Int J Vaccin Vaccination 1: 00012.

[11] Khan MT Zaheer S Shafique K, 2017 Maternal education, empowerment, economic status and child polio vaccination uptake in Pakistan: a population based cross sectional study. BMJ Open 7: e013853. 10.1136/bmjopen-2016-013853 PMC5353333 28283489

[12] Adokiya MN Baguune B Ndago JA, 2017 Evaluation of immunization coverage and its associated factors among children 12–23 months of age in Techiman Municipality, Ghana, 2016. Arch Public Health 75: 28. 28652913 10.1186/s13690-017-0196-6 PMC5483840

[13] Oliveira MF Martinez EZ Rocha JS, 2014 Factors associated with vaccination coverage in children < 5 years in Angola. Rev Saúde Pública 48: 906–915. 26039393 10.1590/S0034-8910.2014048005284 PMC4285837

[14] Khan QH Sinha T Shrivastatva PK Brahmapurkar KP Brahmapurkar VK, 2015 Assessment of immunization status among children aged 12–23 months, at an urban slum area of Jagdalpur city, Bastar. Healthline J 6: 55–60.

[15] National Center for I Respiratory D, 2011 General recommendations on immunization–recommendations of the Advisory Committee on Immunization Practices (ACIP). MMWR Recomm Rep 60: 1–64. 21293327

[16] Fadnes LT Nankabirwa V Sommerfelt H Tylleskar T Tumwine JK Engebretsen IM Group PES, 2011 Is vaccination coverage a good indicator of age-appropriate vaccination? A prospective study from Uganda. Vaccine 29: 3564–3570. 21402043 10.1016/j.vaccine.2011.02.093

[17] Sadoh AE Eregie CO, 2009 Timeliness and completion rate of immunization among Nigerian children attending a clinic-based immunization service. J Health Popul Nutr 27: 391–395. 19507754 10.3329/jhpn.v27i3.3381 PMC2761795

