# Supplementary material for: Improvements in Polio Vaccination Status and Knowledge about Polio Vaccination in the CORE Group Polio Project Implementation Areas in Pastoralist and Semi-Pastoralist Regions in Ethiopia
Source: Am J Trop Med Hyg. 2019 Oct;101(4 Suppl):52–8. doi: 10.4269/ajtmh.19-0022 (PMC6776097; doi:10.4269/ajtmh.19-0022)
Supplement: Supplementary file 1 [file tpmd190022.SD1.docx]

**Supplemental Appendix**

**Supplemental Appendix Table 1. Vaccine-specific coverage and overall coverage by data source and survey**

| **Vaccine dose** | **EDHS 2011** | | **EDHS 2016** | | **CGPP 2013 survey** | | **CGPP 2015 survey** | | | **CGPP 2017 survey** | | |
| --- | --- | --- | --- | --- | --- | --- | --- | --- | --- | --- | --- | --- |
| **OPV 0** | n | %^3^ | n | %^3^ | n | %^3^ | n | %^3^ | | n | %^3^ | |
| Not vaccinated | 804 | 84.6 | 701 | 76.4 | 267 | 51.3 | 253 | 41.1 | 318 | | | 46.2 |
| Card^1^ | 75 | 4.6 | 167 | 10.7 | 114 | 17.8 | 123 | 23.5 | 151 | | | 25.6 |
| History (if no card) | 115 | 10.7 | 152 | 13.0 | 226 | 30.8 | 209 | 35.4 | 203 | | | 28.2 |
| **OPV 1** |  |  |  |  |  |  |  |  |  | | |  |
| Not vaccinated | 222 | 21.3 | 225 | 23.0 | 57 | 8.5 | 207 | 33.0 | 117 | | | 15.8 |
| Card | 212 | 21.0 | 480 | 46.1 | 242 | 44.8 | 356 | 63.7 | 330 | | | 48.7 |
| History (if no card) | 560 | 57.8 | 315 | 30.9 | 308 | 46.7 | 22 | 3.3 | 225 | | | 35.5 |
| **OPV 2** |  |  |  |  |  |  |  |  |  | | |  |
| Not vaccinated | 332 | 34.1 | 322 | 33.3 |  |  | 210 | 33.6 | 137 | | | 18.8 |
| Card | 183 | 18.0 | 446 | 41.0 |  |  | 348 | 62.1 | 318 | | | 47.3 |
| History (if no card) | 479 | 47.9 | 252 | 25.7 |  |  | 27 | 4.3 | 217 | | | 33.9 |
| **OPV 3** |  |  |  |  |  |  |  |  |  | | |  |
| Not vaccinated | 591 | 59.9 | 481 | 50.2 | 88 | 13.6 | 209 | 33.2 | 180 | | | 26.4 |
| Card | 145 | 14.2 | 402 | 35.7 | 211 | 38.6 | 345 | 63.1 | 306 | | | 46.0 |
| History (if no card) | 258 | 25.9 | 137 | 14.0 | 308 | 47.8 | 31 | 3.7 | 186 | | | 27.6 |
| **OPV coverage (card only)** | |  |  |  |  |  |  |  | |  |  | |
| Not vaccinated | 775 | 78.4 | 525 | 52.9 |  |  | 215 | 30.8 | | 332 | 49.4 | |
| Partially | 82 | 8.0 | 106 | 12.4 |  |  | 42 | 7.2 | | 45 | 5.8 | |
| Fully vaccinated | 137 | 13.6 | 389 | 34.7 |  |  | 328 | 62.1 | | 295 | 44.8 | |
| **OPV coverage (card + history)** | |  |  |  |  |  |  |  | |  |  | |
| Not vaccinated | 213 | 20.9 | 225 | 23.0 |  |  | 207 | 32.9 | | 111 | 14.8 | |
| Partially | 387 | 39.9 | 256 | 27.2 |  |  | 4 | 0.8 | | 76 | 12.6 | |
| Fully vaccinated | 394 | 39.5 | 539 | 49.8 |  |  | 374 | 66.3 | | 485 | 72.6 | |
| Age at vaccination for^3^ |  |  |  |  |  |  |  |  | |  |  | |
| OPV 0: > 15 days | 44 | 50.0 | 35 | 27.5 |  |  | 27 | 20.4 | | 18 | 9.5 | |
| OPV 1: < 6 weeks | 37 | 12.8 | 98 | 14.8 |  |  | 88 | 20.4 | | 59 | 17.7 | |
| Interval < 4 weeks between^3^ | |  |  |  |  |  |  |  | |  |  | |
| OPV 1 and OPV 2 | 16 | 5.0 | 53 | 11.3 |  |  | 38 | 11.7 | | 23 | 7.5 | |
| OPV 2 and OPV 3 | 15 | 8.7 | 39 | 10.2 |  |  | 22 | 6.3 | | 28 | 9.0 | |
| Total | 994 | | 1,020 | | 607 | | 585 | | | 672 | | |

^1^ Card refers to information obtained from the child’s immunization card

^2^ Fully vaccinated refers to those with the three doses of OPV (OPV 1, OPV 2 and OPV 3)

Note: EDHS; Ethiopian DHS survey; Shaded areas indicate that no data were available to calculate these statistics.

^3^Percentages are weighted for EDHS 2011 & 2016 whereas for the CGPP surveys adjusted for cluster sampling

**
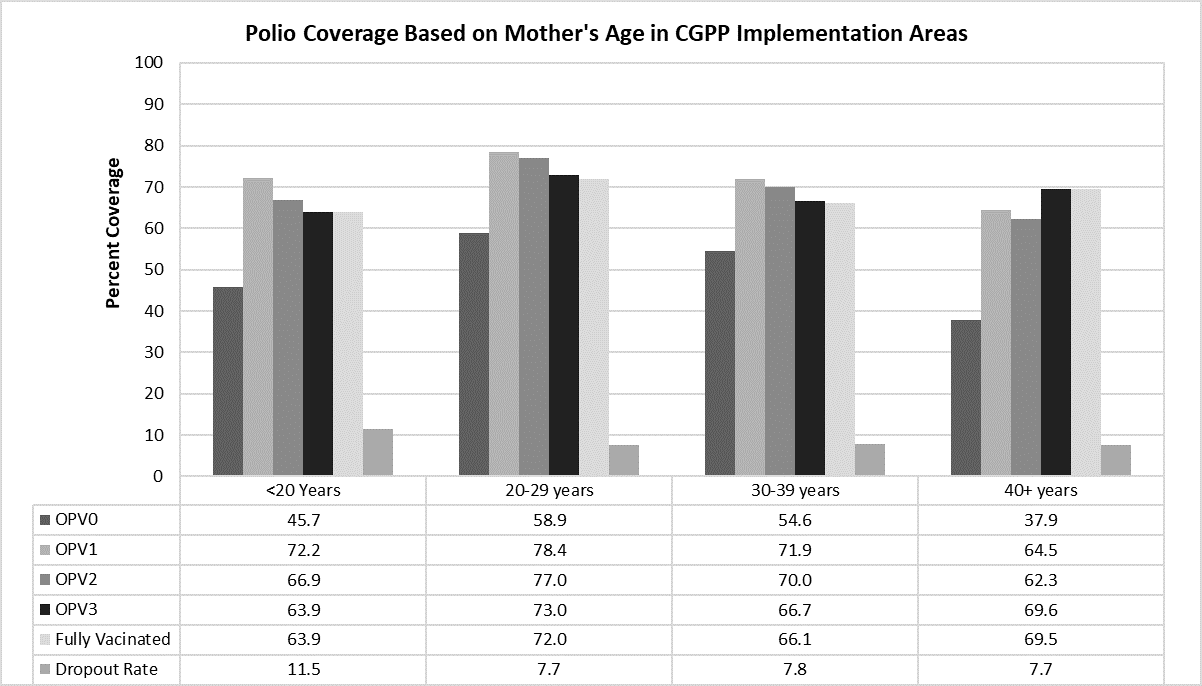
**

**Supplemental Appendix Figure 1: Polio dose-specific and full vaccination coverage (FVC) and dropout rate (DOR) by age of mother in the CGPP implementation areas (2015 and 2017 survey data combined)**

Note: the baseline (2013) survey did not collect data on mother’s age


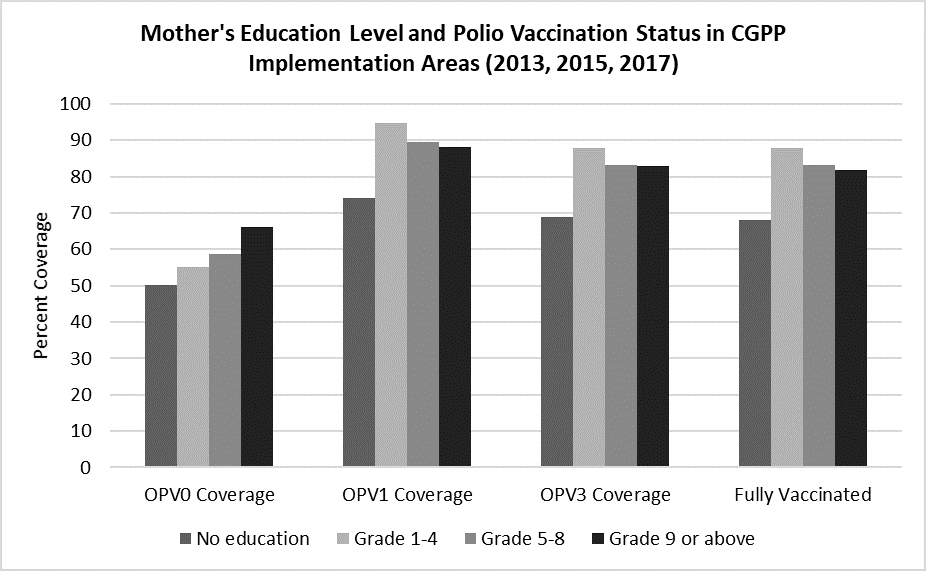


**Supplemental Appendix Figure 2. OPV 0, OPV1, OPV 3, and full vaccination coverage levels by educational status of mother in the CORE Group Polio Project implementation areas (2013, 2015, and 2017 data combined)**

**Supplemental Appendix Table 2. Oral polio vaccination coverage levels by religion of mother in the CORE Group Polio Project implementation area, 2013-2017**

| **Vaccine/Year** | | **Number not vaccinated** | | | **Total** | **Number vaccinated** | | | **Total** | **Percent vaccinated** | | | **Total** |
| --- | --- | --- | --- | --- | --- | --- | --- | --- | --- | --- | --- | --- | --- |
|  |  | **Christians** | **Muslim** | **Other** |  | **Christians** | **Muslim** | **Other** |  | **Christians** | **Muslim** | **Other** |  |
| OPV 0 | 2013 | 89 | 128 | 45 | **262** | 132 | 165 | 40 | **337** | 50.6 | 51.3 | 32.8 | 48.7 |
|  | 2015 | 77 | 154 | 12 | **243** | 168 | 145 | 10 | **323** | 72.6 | 48.2 | 45.9 | 58.9 |
|  | 2017 | 108 | 190 | 20 | **318** | 178 | 157 | 19 | **354** | 71.4 | 45.3 | 48.5 | 53.8 |
| OPV 1 | 2013 | 8 | 30 | 16 | 54 | 213 | 263 | 69 | 545 | 97.8 | 86.4 | 86.2 | 91.5 |
|  | 2015 | 45 | 146 | 10 | 201 | 200 | 153 | 12 | 365 | 90.9 | 50.8 | 55.6 | 67.1 |
|  | 2017 | 35 | 74 | 8 | 117 | 251 | 273 | 31 | 555 | 91.2 | 77.7 | 78.5 | 84.2 |
| OPV 2 | 2013 |  |  |  |  |  |  |  |  |  |  |  |  |
|  | 2015 | 47 | 147 | 10 | 204 | 198 | 152 | 12 | 362 | 90.9 | 50.5 | 55.6 | 66.4 |
|  | 2017 | 43 | 86 | 8 | 137 | 243 | 261 | 31 | 535 | 91.2 | 74.0 | 78.5 | 81.2 |
| OPV 3 | 2013 | 22 | 44 | 20 | 86 | 199 | 249 | 65 | 513 | 91.4 | 82.9 | 79.8 | 86.4 |
|  | 2015 | 47 | 146 | 10 | 203 | 198 | 153 | 12 | 363 | 90.2 | 50.8 | 55.6 | 66.8 |
|  | 2017 | 66 | 103 | 11 | 180 | 220 | 244 | 28 | 492 | 90.0 | 68.9 | 71.4 | 73.6 |

Christians” includes those who report that they are Orthodox, Protestant and Catholic

Others” includes those who report that they hold traditional beliefs and those who report that they have no religion

**Supplemental Appendix Table 3. Knowledge of mothers in CORE Group Polio Project implementation areas about polio and acute flaccid paralysis (AFP), about the effect of multiple OPV doses, and age at first OPV dose, 2013-2017**

| **Background characteristics** | **N** | **Heard about polio or AFP (n=1,858)** | | | **Belief about effect of giving a child multiple OPV doses (n=1,817)** | | | | **Knowledge on timing first OPV dose (n=1,861)** | | | |
| --- | --- | --- | --- | --- | --- | --- | --- | --- | --- | --- | --- | --- |
|  |  | **Yes** | **No** | **p-value** | **More protected** | **May be harmed** | **DK** | **p-value** | **First 2 weeks** | **After 2 weeks** | **DK** | **p-value** |
| CGPP data source |  |  |  | < 0.001 |  |  |  | < 0.001 |  |  |  | 0.002 |
| Baseline 2013 | 603 | 63.8 | 36.2 |  | 68.8 | 8.1 | 23.1 |  | 43.5 | 21.3 | 35.2 |  |
| Mid-term 2015 | 583 | 93.8 | 6.2 |  | 83.7 | 10.8 | 5.5 |  | 57.1 | 30.1 | 12.9 |  |
| Final 2017 | 672 | 92.0 | 8.0 |  | 81.0 | 11.3 | 7.7 |  | 54.2 | 31.1 | 14.8 |  |
| Total | 1858 | 84.7 | 15.3 |  | 78.6 | 10.2 | 11.2 |  | 52.2 | 28.0 | 19.9 |  |
| Residence |  |  |  | 0.101 |  |  |  | 0.055 |  |  |  | 0.001 |
| Rural | 1701 | 83.6 | 16.7 |  | 77.3 | 10.8 | 12.0 |  | 48.9 | 29.7 | 21.5 |  |
| Urban | 157 | 95.4 | 4.6 |  | 91.3 | 4.9 | 3.8 |  | 83.7 | 11.6 | 4.7 |  |
| Total | 1858 | 84.7 | 15.3 |  | 78.6 | 10.2 | 11.2 |  | 52.2 | 28.0 | 19.9 |  |
| Mother's education |  |  |  | < 0.011 |  |  |  | 0.006 |  |  |  | 0.021 |
| No education | 1222 | 83.9 | 16.1 |  | 76.3 | 11.6 | 12.0 |  | 50.9 | 27.6 | 21.5 |  |
| 1-4 grade | 156 | 73.7 | 26.3 |  | 78.1 | 2.5 | 19.4 |  | 51.1 | 18.6 | 30.3 |  |
| 5-8 grade | 252 | 86.4 | 13.6 |  | 81.7 | 9.3 | 9.1 |  | 51.3 | 31.3 | 17.4 |  |
| 9 or above | 228 | 94.3 | 5.7 |  | 85.5 | 10.3 | 4.3 |  | 59.7 | 32.1 | 8.2 |  |
| Total | 1858 | 84.7 | 15.3 |  | 78.6 | 10.2 | 11.2 |  | 52.2 | 28.0 | 19.9 |  |
| Religion |  |  |  | < 0.001 |  |  |  | 0.011 |  |  |  | 0.042 |
| Muslim | 937 | 90.6 | 9.4 |  | 71.5 | 17.3 | 11.2 |  | 53.0 | 29.4 | 17.6 |  |
| Christians | 390 | 71.2 | 28.8 |  | 78.5 | 6.1 | 15.5 |  | 46.1 | 24.1 | 29.8 |  |
| Other | 141 | 71.2 | 28.8 |  | 75.3 | 2.7 | 22.0 |  | 47.5 | 21.5 | 31.0 |  |
| Total | 1468 | 82.7 | 17.3 |  | 74.1 | 12.3 | 13.6 |  | 50.3 | 27.0 | 22.7 |  |
| Child place of birth** | |  |  | 0.153 |  |  |  | < 0.001 |  |  |  | 0.004 |
| Home | 315 | 89.9 | 10.1 |  | 65.2 | 21.1 | 13.7 |  | 46.9 | 32.5 | 20.6 |  |
| Health facility | 350 | 93.9 | 6.1 |  | 93.0 | 3.8 | 3.2 |  | 60.4 | 29.9 | 9.6 |  |
| Total | 665 | 91.9 | 8.1 |  | 80.9 | 11.3 | 7.8 |  | 54.4 | 31.1 | 14.5 |  |

(*) 41 respondents who reported that child not harmed or protected if received repeated OPV are excluded

Note: DK: don’t know (**) Place of birth data is available only for 2017 survey data
